# Mucous secretion in rat colonic mucosa during carcinogenesis induced by dimethylhydrazine. A morphological and histochemical study.

**DOI:** 10.1038/bjc.1975.134

**Published:** 1975-07

**Authors:** M. I. Filipe

## Abstract

**Images:**


*Br. J. Cancer* (1975) **32**, 60

### MUCOUS SECRETION IN RAT COLONIC MUCOSA DURING CARCINOGENESIS INDUCED BY DIMETHYLHYDRAZINE. A MORPHOLOGICAL AND HISTOCHEMICAL STUDY

M. I. FILIPE

*From the Department of Histopathology, Udall Street Laboratories, Westminster Medical School, London SW1P 2PP*

Received 3 March 1975. Accepted 22 March 1975

**Summary.—Our previous studies, in specimens of large intestine resected for carcinoma, have shown abnormal patterns of mucous secretion in areas of apparently "normal" mucosa, where goblet cells produce mainly sialomucins as compared with the true normal colonic mucosa in which sulphomucins predominate. In the present work, large bowel cancer was induced in rats by the administration of 1,2-dimethylhydrazine-2HCl (DMH). We attempted to study the sequential histological and secretory abnormalities which developed in the colonic epithelium during carcinogenesis, and to correlate these changes with those described above in the human. The microscopical and histological lesions observed in the colonic mucosa of DMH treated rats confirmed the findings of other authors and resembled the human colorectal cancer. The earliest changes detected were small foci of hyperplasia accompanied from the 6th week onwards by several foci of dysplasia. Carcinoma *in situ* appeared at the 15th week and finally invasive carcinoma developed from the 19th week onwards. Changes in the type of mucous secretion, with predominance of sialomucins, were observed in the majority of the areas showing mild to moderate dysplasia whilst the surrounding normal epithelium produced sulphated material. Mucous depletion was a common feature in areas of severe dysplasia and carcinoma.**

**These findings correlated well with the similar variations in the mucin composition observed in human colonic mucosa in carcinoma and further supported our previous hypothesis that mucin changes characterized by an increase in sialomucins might reflect early malignant transformation. If this hypothesis proved to be correct, the use of a simple method for the identification of mucins in large bowel biopsies would be of great help in detecting early malignancy.**

IN MAN, the non-neoplastic mucosa adjacent to carcinoma of the large intestine ("transitional" mucosa as we provisionally termed it) is quite often histochemically and ultrastructurally abnormal (Filipe, 1969, 1971*a*; Dawson and Filipe, 1975). It may be normal in haematoxylin–eosin stained sections or may show longer crypts with branching and larger goblet cells distended with mucus (Filipe and Branfoot, 1974). The histochemical changes in the composition of the goblet cell mucin consist of an increase in sialomucins as compared with the normal mucosa in which sulphomucins predominate. The higher level of sialic acids is accompanied by a rise in neuraminidase-sensitive sialic acids (Filipe and Cooke, 1974), a feature described in the human foetal gut mucin (Lev and Orlic, 1974).

From these previous findings, it is possible that the mucin changes described are either secondary to the tumour growth or are a primary cellular response to unknown stimuli (*e.g.* carcinogens) and thus indicative of early malignant transformation.

In an attempt to clarify these points, we studied in detail the mucosa of the entire length of large intestinal specimens resected for carcinoma (Filipe and Branfoot, 1974). We observed that mucin changes with a predominance of sialomucins were present not only in the apparently normal mucosa adjacent to the carcinoma but also in patches of normal mucosa at various distances from the tumour. These observations suggest that the modifications in the secretory product may represent an early feature of malignancy.

To elucidate this problem further, it is of the utmost importance to use an animal model for colorectal carcinoma. The present work reports our findings in rats in which neoplasms of the large intestine were selectively induced by 1,2-dimethylhydrazine-2HCl (Druckrey *et al.*, 1967; Druckrey, 1970).

Our purpose was to investigate the morphological and mucin changes occurring in the rat colonic epithelium, with particular emphasis on the early stages of carcinogenesis, and also to determine whether changes in the mucin composition in the rat are similar to those described in the human colonic mucosa in cases with carcinoma.

### MATERIALS AND METHODS

*Animal experiments.*—75 white Wistar female rats (A. Tuck & Son Ltd, The Nursery, Rayleigh, Essex, England) weighing approximately 140 g at the beginning of the experimental period were used in this study. The animals were fed with standard diet 41B and water *ad libitum*. The "test" rats (50) were injected subcutaneously with a weekly dose of 20 mg/kg body weight, expressed as the base of 1,2-dimethylhydrazine-2HCl (DMH). The DMH solution was prepared according to Wiebecke *et al.* (1969) modified by P. N. Magee (personal communication) as follows: the 1,2-dimethylhydrazine-2HCl (Ralph Emanuel Ltd, Wembley, Middlesex, England) was freshly dissolved in 0·9% NaCl containing 1·5% EDTA to give a 0·35% solution with respect to the base and the pH was adjusted to 6·5 with 1% NaOH. The control animals (25) were given a weekly subcutaneous injection of 0·9% NaCl containing 1·5% EDTA. The experiment lasted for 29 weeks.

*Preparation of tissues.*—Groups of 3 rats (2 tests and 1 control) were killed each week from the start of the experiment. The whole of the large intestine was removed, opened, pinned down onto cork and fixed in 10% neutral formol-calcium. After fixation, the entire specimen was divided into lengths and coiled up into "swiss-rolls" (Fig. 1). Blocks were also taken when the presence of large neoplasms made the use of "swiss-rolls" impractical.

All tissue was processed routinely and embedded in paraffin. Not less than 4 serial sections were cut at 5 μm thickness. Blocks containing tumours were sectioned at 3 or more different levels and serial sections were taken, as above, from each level. The sections were stained with haematoxylin and eosin (H. and E.) and by the following histochemical techniques for the identification of mucins: periodic acid-Schiff (PAS) (Pearse, 1968), high-iron-diamine (HID) and high-iron-diamine-Alcian blue (HID-AB) for the visualization of both sialo- and sulphated mucins (Spicer, 1965; Gad and Sylven, 1969; Sorvari, 1972).

*Mapping of morphological changes and mucin distribution.*—The images of each of the H. and E. and HID-AB stained serial sections were projected onto paper and tracings were made of the mucosal outlines (Fig. 2). Areas showing alterations in the morphology and in the mucin composition were recorded on the graphs so that we could obtain an accurate chart correlating the histological features and the patterns of mucous secretion (Fig. 1, 2).

### RESULTS

During all stages of the experimental period in which the rat colonic mucosa is subjected to the effect of the carcinogen, a wide spectrum of histological and histochemical (mucous secretion) modifications occur. The lesions are more severe and extensive in the distal colon and rectum than in the proximal colon and caecum.

### *Macroscopy*

Caecum, proximal and distal colon can easily be distinguished by the different arrangement of the mucosal folds. In the caecum the folds are prominent and

Rat Left Colon: Distribution of Macroscopical, Histological and Mucin Changes during Carcinogenesis

Each column represents one DMH treated rat. Each symbol = one focal lesion, secreting either sulphomucins (○), or sialomucins (●) or both (◐). △ = Mucous secretion absent or scanty (△ sialo- or sulphomucins ▲). □ = macroscopical lesions.

** = Generalized dysplasia.

*** = Superficial dysplasia.

at varying angles whilst in the proximal colon they display a "herring-bone" pattern. In the distal segment the mucosa is often smooth with the folds running longitudinally.

In the controls no macroscopical changes were observed in the mucosa, during the 29 weeks duration of the experiment.

In the tests no recognizable lesions are detected up to 12 weeks (Table I). At the 13th week one of the tests showed 2 small excrescences in the transitory distal-proximal colon (at 15 and 15·5 cm from the anus respectively). With light microscopy they represent areas of dysplasia. Other small mucosal protrusions are noted in the distal colon at the 15th week, one of them being histologically an area of carcinoma *in situ*.

The number and size of the macroscopical lesions increase with longer exposure to DMH (Table I) and from the 19th week onwards all rats develop neoplasms either as plaques or as sessile polyps measuring between 0·3 and 0·8 mm in diameter.

Of the 54 macroscopically recognizable lesions found between the 19th and 28th weeks of the experiment, 29 are invasive carcinoma, out of which 79% are located in the distal colon.

*Histology* (Table I)

*Controls*.—The distal colon reveals similar histological features to the human colorectal mucosa, with straight crypts lined by mucus secreting goblet cells (Fig. 3). The goblet cells predominate in the $\frac{2}{3}$ upper crypt and are interspersed

FIG. 3.—Control rat. Distal colon. HID-AB staining to show the predominance of dark coloured sulphomucins. ×600.

by absorptive cells. In the proximal colon, the crypts are branched and the goblet cells and absorptive cells are present in the $\frac{2}{3}$ or $\frac{1}{2}$ upper crypt, whilst the lower compartment is occupied by a further type of epithelial cell. This third type of cell is globular, with a vast pale cytoplasm in H. and E., and producing mucus which differs histochemically from the secretory product of the goblet cells (Fig. 4) (see Histochemistry section and Table II).

In this group of animals, no histological abnormalities other than the occasional mild oedema in the lamina propria are demonstrated.

*Tests.*—The morphological lesions observed, involving 1, 2 or more glandular tubules, may be grouped into 4 categories according to the degree of histological dedifferentiation (Muto and Morson, 1975) and defined as follows:

(1) *Epithelial hyperplasia:* Where the crypts are longer, the lumen may be dilated and the goblet cells are more numerous, taller, and distended with mucus. The nuclei are aligned regularly on the basement membrane.

(2) *Dysplasia* is graded 0–2. In grade 0 (Fig. 5) the crypts are not usually longer but may be dilated, the goblet cells are globular and between them the

FIG. 4.—Control rat. Proximal colon. HID-AB Technique to show the predominance of sialomucins (grey) in the $\frac{2}{3}$ lower crypt; a few goblet cells containing sulphated material (black) may be seen in the upper crypt. The HID-AB method stains sialomucins blue (grey), whilst sulphomucins are stained brown-black (black). ×600.

TABLE II.—*Histochemical Characteristics of the Mucins in the Various Segments of the Rat Large Intestine*

| | Distal colon | | Proximal colon | | Caecum | |
|---|---|---|---|---|---|---|
| | PAS | HID-AB | PAS | HID-AB | PAS | HID-AB |
| ⅓ upper crypt | +++ | Brown | +/++/+++ | Blue/brown | +++ | Brown |
| Middle crypt | +++ | Brown | +/++ | Blue | +++ | Brown |
| ⅓ lower crypt | +++ | Brown | +/++ | Blue | ++ | Brown/blue |

FIG. 5.—DMH treated rat. Grade 0 dysplasia in a single gland. The HID-AB method shows that the atypical gland (arrow) produces sialomucins (grey) compared with the surrounding glands secreting sulphomucins (black). ×600.

absorptive cells have a more eosinophilic ground-glass cytoplasm. These features are more commonly noted in the upper half of the crypt. In grade 1 (Fig. 6 ABC) there is a distortion of the crypts' contours, hyperchromatic nuclei, moderate pseudostratification but with the preservation of nuclear polarity, and a slight reduction in the number of goblet cells. Mitotic figures are not present.

As it is not rare to find two grades of atypism in the same focus of dysplasia, or even in the same crypt, we will group them together under grade 0–1 (Fig.

Fig. 6A

Fig 6B

FIG. 6C

FIG. 6.—DMH treated rat. Grade 1 dysplasia. (A) H. and E. (B) HID to show a marked decrease in sulphomucins in the area of dysplasia, whilst sialomucins are predominant as shown by the HID-AB (C). ×300.

7A, B). Grade 1 (Table I) groups foci consisting of crypts of grade 1 only; grade 2 groups one or more crypts showing marked hyperchromatism, loss of nuclear polarity and mitotic figures. Also, tubular irregularity is common, goblet cells are rare and thus mucus production is very much reduced. Quite often, grade 1 and 2 can be demonstrated in one focus of dysplasia and it is easier to put them together in grade 1–2 (Fig. 8A, B).

(3) *Carcinoma* in situ (Fig. 9A, B) corresponds to the severe dysplasia grade 3 of Muto and Morson (1975). The crypts are tortuous and the nuclei larger, hyperchromatic and located at differing heights in the cell. Mitotic figures are present. There is no mucus production and goblet cells are either reduced to a tiny " goblet " close to the crypt lumen or more often are absent. Carcinoma *in situ* may be demonstrated in as small a group as 3–4 tubules either in a flat mucosa or in a polypoid lesion.

(4) *Invasive carcinoma* (Fig. 10): There is severe dysplasia with definite evidence of submucosal invasion. The tumours are then classified into grades I–IV of histological dedifferentiation according to Dukes (1940).

Both carcinoma *in situ* and invasive carcinoma are often surrounded by areas of hyperplasia and tubules showing various degrees of dysplasia (Fig. 9A, B).

During the first 3 weeks of the experiment (Table I), no histological changes are detected. In the following 3 weeks

FIG. 7A

FIG. 7B

FIG. 7.—DMH treated rat. Grade 0–1 dysplasia. (A) H. and E. ×600. (B) HID-AB revealing the presence of sialomucins (blue) in the area of dysplasia, surrounded by normal glands producing sulphomucins (brown). ×150.

FIG. 8A

FIG 8B

FIG. 8.—DMH treated rat. Grade 1-2 dysplasia. (A) H. and E. ×600. (B) HID-AB showing the presence of sialomucins (blue) in the area of dysplasia, whilst sulphomucins (brown) predominate in the normal surrounding glands. ×150.

foci of hyperplasia are the only findings, with an occasional crypt showing mild or rarely severe dysplasia. From the 7th to the 13th week the number and severity of the histological lesions increase. During this period hyperplasia and all grades of dysplasia are observed. However, foci with severe atypia (grade 2) are still uncommon. From the 13th week onwards (Table I) the number of focal areas showing severe dysplasia (grade 2) increases markedly and histological features of carcinoma *in situ* appear. Invasive carcinoma is first demonstrated in rats killed after receiving 19 injections of DMH and in all rats with longer periods of treatment, except in one case where 5 areas of carcinoma *in situ* appeared in the mucosa but no invasive carcinoma was detected.

All the carcinomata found in the distal colon are well differentiated adeno-

FIG. 9A

FIG 9B

FIG. 9.—DMH treated rat. Carcinoma *in situ* (T) surrounded by an area of hyperplasia and dysplasia. (A) HID-AB-sialomucins (grey) are the mainly secretory product in the areas of dysplasia accompanied by a decrease in sulphated material as shown in section (B) stained by HID. Areas of hyperplasia contain predominantly sulphomucins (black). Note absence of mucous secretion in the area of carcinoma *in situ*. $\times 600$.

FIG. 10.—DMH treated rat. Well differentiated adenocarcinoma in a flat mucosa (endophytic growth). H. and E. ×180.

carcinomata (grade I), a few of them showing " endophytic " growth (Fig. 10). Three of 5 invasive carcinomata in the proximal colon produced abundant mucus and presented features of " signet ring " carcinoma whilst the other 2 were grade 1.

*Mucous secretion* (*histochemistry*)

*Controls.*—The composition and distribution of the epithelial mucins vary in the different segments of the colon and caecum (Table II). In the distal colon, the histochemical characteristics of the goblet cell mucins and their distribution in the crypt epithelium are similar to the human colonic mucosa. The goblet cells located along the whole crypt or in the $\frac{2}{3}$ lower part contain mainly sulphomucins (Fig. 3). Sialomucins may be present in the goblet cells of the upper crypt and surface epithelium. They also show a strong to moderate PAS reactivity. This pattern changes in the proximal colon, where sialomucins are the predominant content of the mucus secreting cells all along the crypt epithelium. Occasional goblet cells in the upper crypt and surface epithelium may show sulphated material (Fig. 4). The goblet cells of the upper crypt and surface epithelium have a strong to moderate PAS reaction whereas the mucus secreting cells in the lower crypt react weakly with PAS.

The transition from distal to proximal colon is gradual, sialomucins being present in the lower half of the crypt whereas in the upper half sulphomucins predominate.

In the caecum, goblet cells are distributed in reduced numbers from the middle crypt upwards and their contents have the staining properties of the sulphomucins, whilst in the bottom of the crypt both types of acid mucosubstances may be present.

Owing to the morphological and histochemical similarities between the rat distal colon and the human colorectal mucosa, we will refer only to the changes observed in the distal colon which may be accepted as a suitable experimental model for the present work.

*Tests.*—The rats treated with DMH develop changes in the mucin composition. These consist of a predominance of sialomucins in the goblet cells, accompanied by a decrease or absence of sulphomucins, similar to those changes described in the human colonic mucosa in specimens with carcinoma. These modifications in the secretory product correlate well with the changes in the morphology.

Based on the relationship between mucin changes and focal areas of dysplasia, we may consider 3 or possibly 4 stages in the process of chemically induced carcinogenesis in the rat, as follows (Table I):

A. The mucosa in the first 6 weeks after the initiation of the treatment is either histologically normal or may show areas of hyperplasia. No alteration is observed in the mucin composition even in the areas of hyperplasia where goblet cells contain sulphomucins as in the controls.

B. From the 7th to 18th week, the number and severity of the lesions gradually increase and this increase is accompanied in general by marked qualitative mucin changes. Again, sulphomucins persist in the hyperplastic glands whereas goblet cells, in the majority of the areas with dysplasia (grade 0–1 and 1), produce mainly sialomucins (Fig. 5–8). Secretion is markedly reduced in grade 2 dysplasia and in the presence of a carcinoma *in situ* (Fig. 9A, B). In both, either sialo- or sulpho- or both types of acid mucins can be detected in the secretory product. During this period of time no carcinoma had yet developed in any of the rats.

C. The period between the 19th and 27th week is characterized histologically by the presence of invasive carcinoma and a greater number of areas with carcinoma *in situ* and severe dysplasia (grade 2). Areas of mild dysplasia are less frequent.

Mucin changes are not commonly seen and, with a few exceptions, foci of dysplasia show either sulphomucins or a mixture of both types of acid mucosubstances. In the distal colon all but 2 carcinomata do not show secretion, or else it is scanty. In one mucus secreting carcinoma there is abundant mucus rich in sialomucins; in the other, only moderate amounts of both sulpho- and sialomucins are present. In the proximal colon 3 " signet ring " carcinomata secreted a mixture of acid mucins (Fig. 11).

D. From the 28th week onwards the mucosa shows the same histological aspects as above but changes in mucin composition are more frequently demonstrated in a greater number of areas of dysplasia (grade 0–1 and 1).

### DISCUSSION

The macroscopical and histological lesions we observed in the colonic mucosa of rats treated with DMH are similar to those described by several other authors (Druckrey *et al.*, 1967; Schauer, Völlnagel and Wildanger, 1969; Druckrey, 1970; Deschner, 1974; Ward, 1974) and resemble the human colorectal cancer, thus providing a suitable animal model for the study of various aspects of carcinogenesis.

The sequential changes occurring in the rat colonic mucosa were followed weekly for a period of 29 weeks and our observations correlate well with the features reported in a similar stepwise investigation carried out in mice by Thurnherr *et al.* (1973) and Deschner (1974). The earlier changes detected at the 4th week are focal areas of hyperplasia in which sulphomucins still predominate as in the normal control musoca. As the experiment progresses the number of lesions with severe dysplasia increases, with carcinoma *in situ* developing at the 15th week and invasive carcinoma appearing at the 19th week. The areas of mild or moderate dysplasia show marked changes in the composition of the goblet cell mucin, characterized by the pre-

FIG. 11.—DMH treated rat. Proximal colon. " Signet " ring carcinoma secreting a mixture of sialo- and sulphomucins. PAS. ×300.

dominance of sialomucins and accompanied in general by a decrease or absence of sulphated material. Studies with $^{35}S$, in fact, show decreased isotope uptake in the non-neoplastic mucosa of rats treated with DMH (Springer, Springer and Oehlert, 1970; Filipe, in preparation) and also in the " normal " mucosa adjacent to colorectal carcinoma in man (Filipe, 1971*b*). It is of interest to note that similar qualitative changes of the epithelial glycoproteins have been found in the human large intestine not only in the " normal " mucosa around carcinoma but also in patches of " normal " mucosa far from the tumour (Filipe, 1969, 1971*a*; Filipe and Branfoot, 1974).

It is not yet possible to answer whether these modifications in the secretory product, in the human, represent an early feature of malignancy. The numerous genetical, physiological and environmental factors which may play a role in human carcinogenesis, as well as other unknown stimuli to which the colonic mucosa is exposed continuously, make it difficult to assess the " specificity " of the mucin changes in the human colonic mucosa. However, our present data from a rat model strongly suggest a relationship between the variations in the mucin composition and the process leading to malignancy, rather than these being the result of other stimuli (Smith and Butler, 1974) or of the toxic effects of DMH (Haase *et al.*, 1973). The mechanism of action of DMH is not yet fully understood but it may interact with nucleic acids and/or protein (Miller and Miller, 1966; Shank and Magee, 1967; Farber, 1968) and thus alter the normal process of cell differentiation and maturation. Indeed, Lipkin and co-workers have described changes in the nucleic acid metabolism and proliferative capacity of the colonic epithelium in DMH treated mice (Thurnherr *et al.*, 1973; Deschner, 1974) which are similar to those found in the human colonic mucosa adjacent to carcinoma and neoplastic polyps (Immondi, Balis and Lipkin, 1969; Deschner and Lipkin, 1970; Troncale, Hertz and Lipkin, 1971) and in familial polyposis (Lipkin, 1974). These changes may reflect a loss of suppressor genes and a regression of the cell to a more embryonic state, a hypothesis supported in the demonstration of embryonic specific antigens in malignant tumours (Gold and Freedman, 1965; von Kleist and Burtin, 1969; Stonehill and Bendich, 1970; von Kleist, 1971; Bordes, Michiels and Martin, 1973).

Whether the raised levels of sialomucins in the colonic epithelium are considered as an expression of cell immaturity or as a result of a direct effect of the carcinogen on the mechanism(s) of the glycoprotein synthesis, and what effect it has on carcinogenesis, we do not know. However, there is cumulative biochemical evidence that the carbohydrate metabolism is profoundly altered in malignantly transformed cells, and sialic acids may play an important role. The presence of sialic acids in the glycoproteins of the cell membrane seem to confer certain properties to the membrane expressed in cell adhesion (Deman, Bruyneel and Mareel, 1974), intercellular contact (Emmelot, 1973), masking antigens (Currie and Bagshawe, 1968; Rios and Simmons, 1973) and changes in its content may be related to the difference in behaviour of normal and malignant cells. Increase in sialic acids and sialyltransferases have been found in malignant cells (Warren, Fuhrer and Buck, 1972; Emmelot, 1973). Changes in glycosyltransferases, as they have been described in malignant cells, may also alter cell contact and the mechanism controlling cell growth and differentiation (Emmelot, 1973). Higher levels of glycosidases have also been reported in the "normal" colonic mucosa and tumours in animals treated with DMH, as compared with controls (Mian and Cowen, 1974; Mian, Ccwen and Nutman, 1974).

It has been suggested (Currie and Bagshawe, 1968; Rios and Simmons, 1973) that tumour cells are coated with neuraminidase sensitive sialic acids which may not only hide their antigens from the host but also shield them from his immunocompetent cells. If this hypothesis is correct, then they may play an important role in the immunological reaction to tumour specific antigens in the control of tumour growth (Baldwin, 1970). In fact, we have found greater amounts of neuraminidase sensitive neuraminic acids in the mucosa around carcinoma in man (Filipe and Cooke, 1974) and they are more abundant in our most invasive tumours (Filipe and Branfoot, 1974). In DMH treated rats we have also observed that the amount of sialomucins present in the focal areas of dysplasia varies during the progress of the experiment, possibly reflecting the immunological status of the host and thus affecting the tumour growth potential.

The changes in the type of glycoprotein secreted by the colonic goblet cells in DMH treated rats further support our previous hypothesis that the increase in sialomucins in the apparently normal mucosa in specimens with carcinoma may represent an early feature of carcinogenesis. At present there is no method which enables us to detect early malignant changes. We feel, therefore, that further investigation of glycoproteins may prove valuable in the search for such a method.

I wish to thank Dr Penelope Dawson and the Photographic Department for their assistance with the photographic work; Mrs J. Hepple for the technical help and Miss L. Turner for typing the manuscript. This work was supported by a grant from the GDF of the Westminster Hospital.

### REFERENCES

Baldwin, R. W. (1970) Tumour Specific Antigens associated with Chemically Induced Tumours. *Rev. Eur. Étud. clin. Biol.*, **15**, 593.

BORDES, M., MICHIELS, R. & MARTIN, F. (1973) Detection of Immunofluorescence of Carcinoembryonic Antigen in Colonic Carcinoma, other Malignant or Benign Tumours and Non-cancerous Tissue. *Digestion*, **9**, 106.

CURRIE, G. A. & BAGSHAWE, K. D. (1968) The Role of Sialic Acid in Antigenic Expression: further Studies of the Landschütz Ascites Tumour. *Br. J. Cancer*, **22**, 843.

DAWSON, P. A. & FILIPE, M. I. (1975) Colonic Epithelium in Colo-rectal Cancer; Ultrastructural and Histochemical Features as Compared with the Normal. Submitted for publication.

DEMAN, J. J., BRUYNEEL, E. A. & MAREEL, M. M. (1974) A Study on the Mechanism of Intercellular Adhesion. Effects of Neuraminidase, Calcium and Trypsin on the Aggregation of Suspended HeLa Cells. *J. cell. Biol.*, **60**, 641.

DESCHNER, E. E. (1974) Experimentally Induced Cancer of the Colon. *Cancer, N.Y.*, **34**, 824.

DESCHNER, E. E. & LIPKIN, M. (1970) Study of Human Rectal Epithelial Cells *in vitro*. III. RNA, Protein and DNA Synthesis in Polyps and Adjacent Mucosa. *J. natn. Cancer Inst.*, **44**, 175.

DRUCKREY, H. (1970) Production of Colonic Carcinomas by 1,2-Dialkylhydrazines and Azoxyalkanes. In *Carcinoma of the Colon and Antecedent Epithelium.* Ed. W. J. Burdette. Springfield, Ill.: C. C. Thomas. p. 267.

DRUCKREY, H., PREUSSMANN, R., MATZKIES, F. & IVANKOVIC, S. (1967) Selektive Enzeugung von Darmkrebs bey Ratten durch 1,2-Dimethylhydrazine. *Naturwissenschaften*, **54**, 285.

DUKES, C. E. (1940) Cancer of the Rectum—An Analysis of 1000 Cases. *J. Path. Bact.*, **50**, 527.

EMMELOT, P. (1973) Biochemical Properties of Normal and Neoplastic Cell Surfaces; A Review. *Eur. J. Cancer*, **9**, 319.

FARBER, E. (1968) Biochemistry of Carcinogenesis. *Cancer Res.*, **28**, 1859.

FILIPE, M. I. (1969) Value of Histochemical Reactions for Mucosubstances in the Diagnosis of Certain Pathological Conditions of the Colon and Rectum. *Gut*, **10**, 577.

FILIPE, M. I. (1971*a*) The Mucous Membrane of the Normal Human Large Intestine and the Changes which Occur in it Immediately Adjacent to Proven Carcinoma—A Histochemical, Autoradiographic and Chemical Study. *Ph.D. Thesis, University of London.*

FILIPE, M. I. (1971*b*) $^{35}$Sulphur Uptake in the Mucosa Adjacent to Carcinoma of the Large Intestine. *Histochem. J.*, **3**, 27.

FILIPE, M. I. & BRANFOOT, A. C. (1974) Abnormal Patterns of Mucus Secretion in Apparently Normal Mucosa of Large Intestine with Carcinoma. *Cancer, N.Y.*, **34**, 282.

FILIPE, M. I. & COOKE, B. K. (1974) Changes in Mucin Composition in the Mucosa Adjacent to Carcinoma of the Colon as Compared with the Normal—A Biochemical Investigation. *J. clin. Path.*, **27**, 315.

GAD, A. & SYLVEN, B. (1969) On the Nature of the High-iron Diamine Method for Sulfomucins. *J. Histochem. Cytochem.*, **17**, 156.

GOLD, P. & FREEDMAN, S. O. (1965) Demonstration of Tumour-specific Antigens in Human Colonic Carcinomata by Immunological Tolerance and Absorption Technique. *J. exp. Med.*, **121**, 439.

HAASE, P., COWEN, D. M., KNOWLES, J. C. & COOPER, E. H. (1973) Evaluation of Dimethylhydrazine Induced Tumours in Mice as a Model System for Colorectal Cancer. *Br. J. Cancer*, **28**, 530.

IMONDI, A. R., BALIS, M. E. & LIPKIN, M. (1969) Changes in Enzyme Levels Accompanying Differentiation of Intestinal Epithelial Cells. *Expl cell Res.*, **58**, 323.

KLEIST, S. VON (1971) Étude d'un antigene specifique des tumeurs coliques humaines d'origine embryonnaire. *Biol. Med.*, **60**, 237.

KLEIST, S. VON & BURTIN, P. (1969) Isolation of a Fetal Antigen from Human Colonic Tumors. *Cancer Res.*, **29**, 1961.

LEV, R. & ORLIC, D. (1974) Histochemical and Autoradiographic Studies of Normal Human Fetal Colon. *Histochemistry*, **39**, 301.

LIPKIN, M. (1974) Phase 1 and Phase 2 Proliferative Lesions of Colonic Epithelial Cells in Diseases Leading to Colonic Cancer. *Cancer, N.Y.*, **34**, 878.

MIAN, N. & COWEN, D. M. (1974) Glycosidases in Normal and Dimethylhydrazine-treated Rats and Mice with Special Reference to the Colonic Tumours. *Br. J. Cancer*, **29**, 438.

MIAN, N., COWEN, D. M. & NUTMAN, C. A. (1974) Glycosidases Heterogeneity among Dimethylhydrazine Induced Rat Colonic Tumours. *Br. J. Cancer*, **30**, 231.

MILLER, E. C. & MILLER, J. A. (1966) Mechanisms of Chemical Carcinogenesis: Nature of Proximate Carcinogens and Interactions with Macromolecules. *Pharmacol. Rev.*, **18**, 805.

MUTO, T. & MORSON, B. C. (1975) Evolution of the Cancer of the Colon and Rectum. *Cancer, N.Y.* In the press.

PEARSE, A. G. E. (1968) *Histochemistry Theoretical and Applied.* 3rd edn. Vol. I. London: Churchill Livingstone.

RIOS, A. & SIMMONS, R. L. (1973) Immunospecific Regression of Various Syngeneic Mouse Tumours in Response to Neuraminidase-treated Tumor Cells. *J. natn. Cancer Inst.*, **51**, 637.

SCHAUER, A., VÖLLNAGEL, TH. & WILDANGER, F. (1969) Cancerisierung des Rattendarmes durch 1,2-Dimethylhydrazin. *Z. ges. exp. Med.*, **150**, 87.

SHANK, R. C. & MAGEE, P. N. (1967) Similarities between the Biochemical Actions of Cycasin and Dimethylnitrosamine. *Biochem. J.*, **105**, 521.

SMITH, B. & BUTLER, M. (1974) The Autonomic Control of Colonic Mucin Secretion in the Mouse. *Br. J. exp. Path.*, **55**, 615.

SORVARI, T. E. (1972) Histochemical Observations on the Role of Ferric Chloride in the High-iron Diamine Technique for Localizing Sulphated Mucosubstances. *Histochem. J.*, **4**, 193.

SPICER, S. S. (1965) Diamine Methods for Differentiating Mucosubstances Histochemically. *J. Histochem. Cytochem.*, **13**, 211.

SPRINGER, P., SPRINGER, J. & OEHLERT, W. (1970) Die Vorstufen des 1,2-Dimethylhydrazine—Unduzierten Dick—und Dunndarmcarcinoms der Ratte. *Z. Krebsforsch.*, **74**, 236.

STONEHILL, E. H. & BENDICH, A. (1970) Retrogenic Expression: The Reappearance of Embryonal Antigens in Cancer Cells. *Nature, Lond.*, **228**, 370.

THURNHERR, N., DESCHNER, E. E., STONEHILL, E. H. & LIPKIN, M. (1973) Induction of Adenocarcinomas of the Colon in Mice by Weekly Injections of 1,2-Dimethylhydrazine. *Cancer Res.*, **33**, 940.

TRONCALE, F., HERTZ, R. & LIPKIN, M. (1971) Nucleic Acid Metabolism in Proliferating and Differentiating Colonic Cells of Man and in Neoplastic Lesions of the Colon. *Cancer Res.*, **31**, 463.

WARD, J. M. (1974) Morphogenesis of Chemically Induced Neoplasms of the Colon and Small Intestine in Rats. *Lab. Invest.*, **30**, 505.

WARREN, L., FUHRER, J. P. & BUCK, C. A. (1972) Surface Glycoproteins of Normal and Transformed Cells: a Difference Determined by Sialic Acid and Growth Dependent Sialyltransferases. *Proc. natn. Acad. Sci. U.S.A.*, **69**, 1838.

WIEBECKE, B., LÖHRS, N., GIMMY, J. & EDER, M. (1969) Erzeugung von Darmtumoren bei Mausen Durch 1,2-Dimethylhydrazin. *Z. Ges. exp. Med.*, **149**, 277.


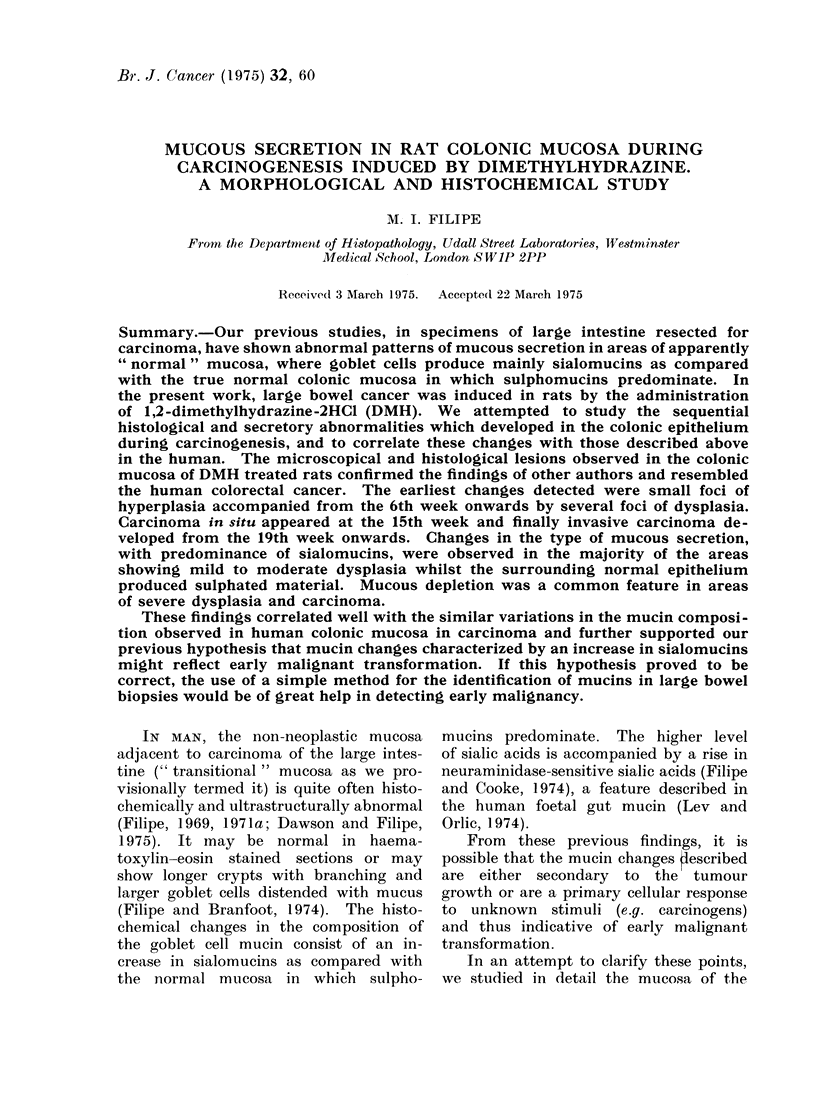

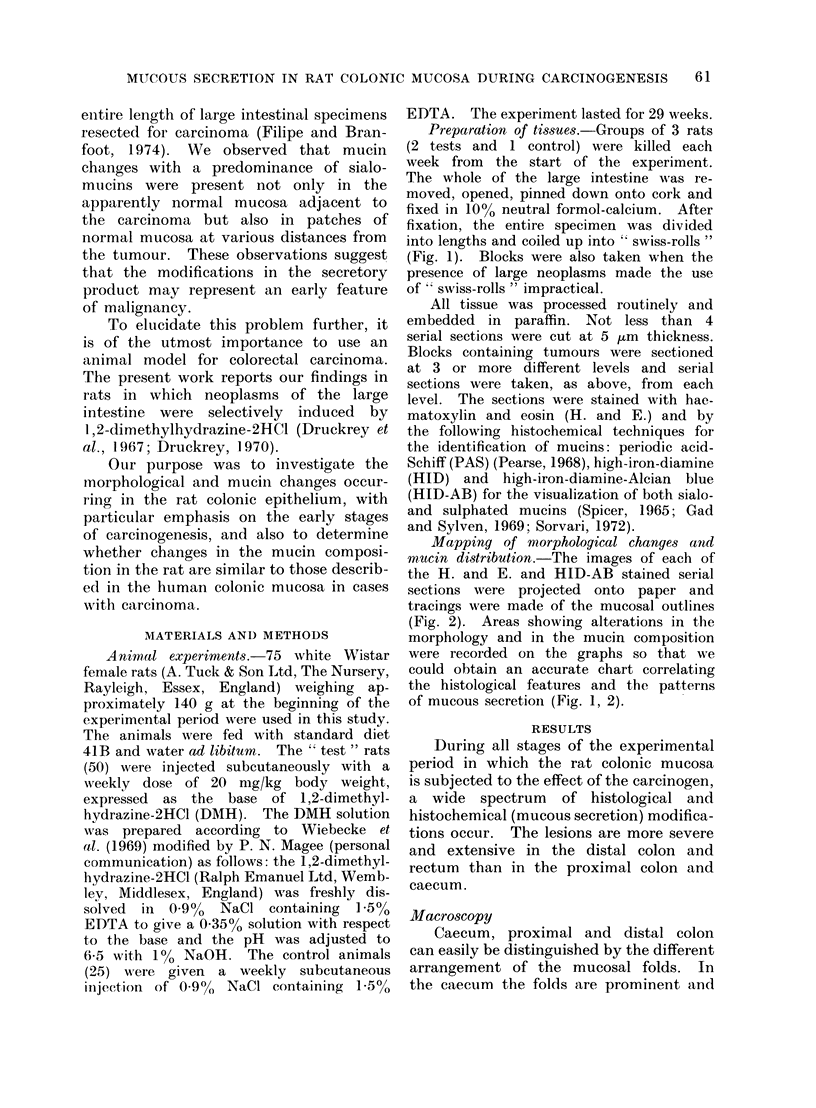

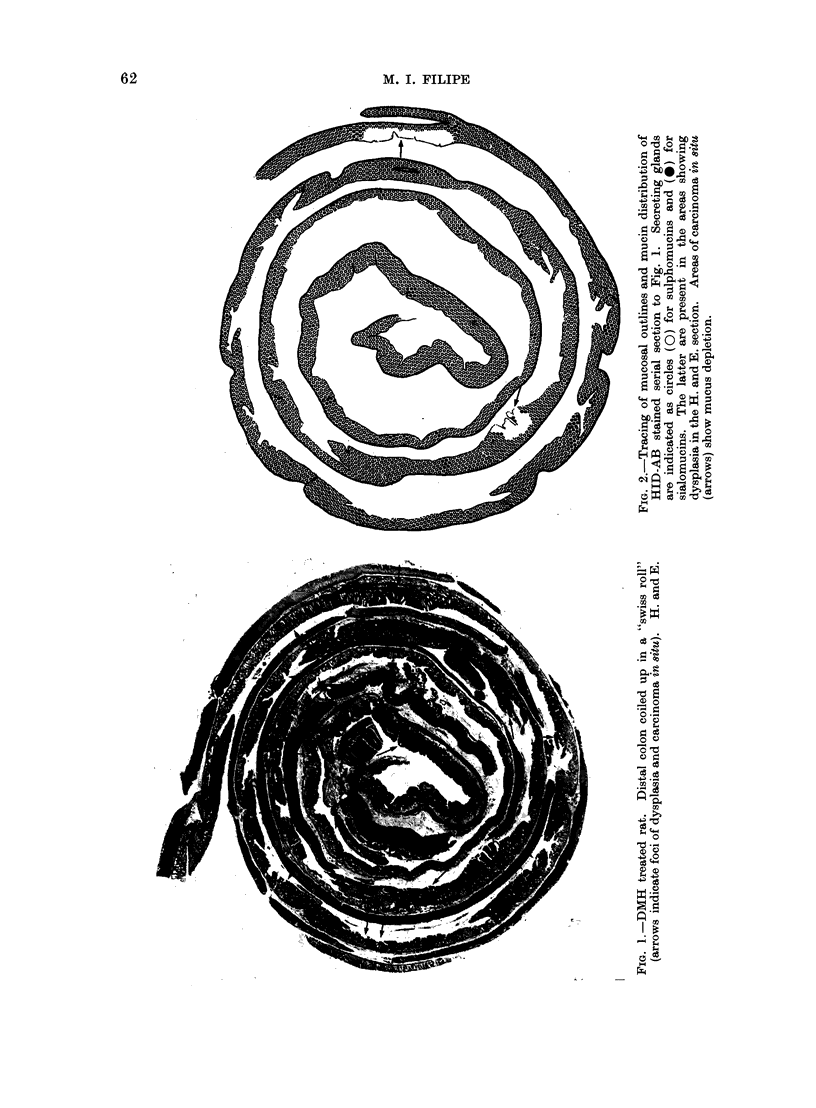

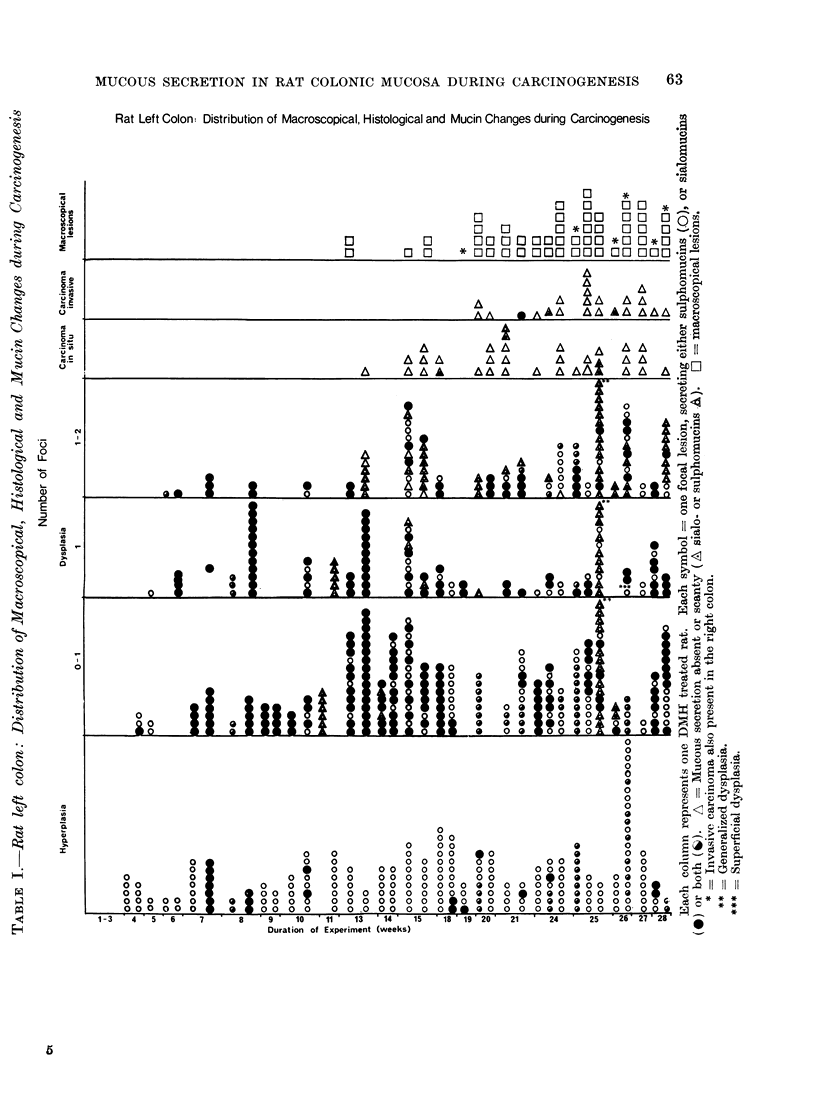

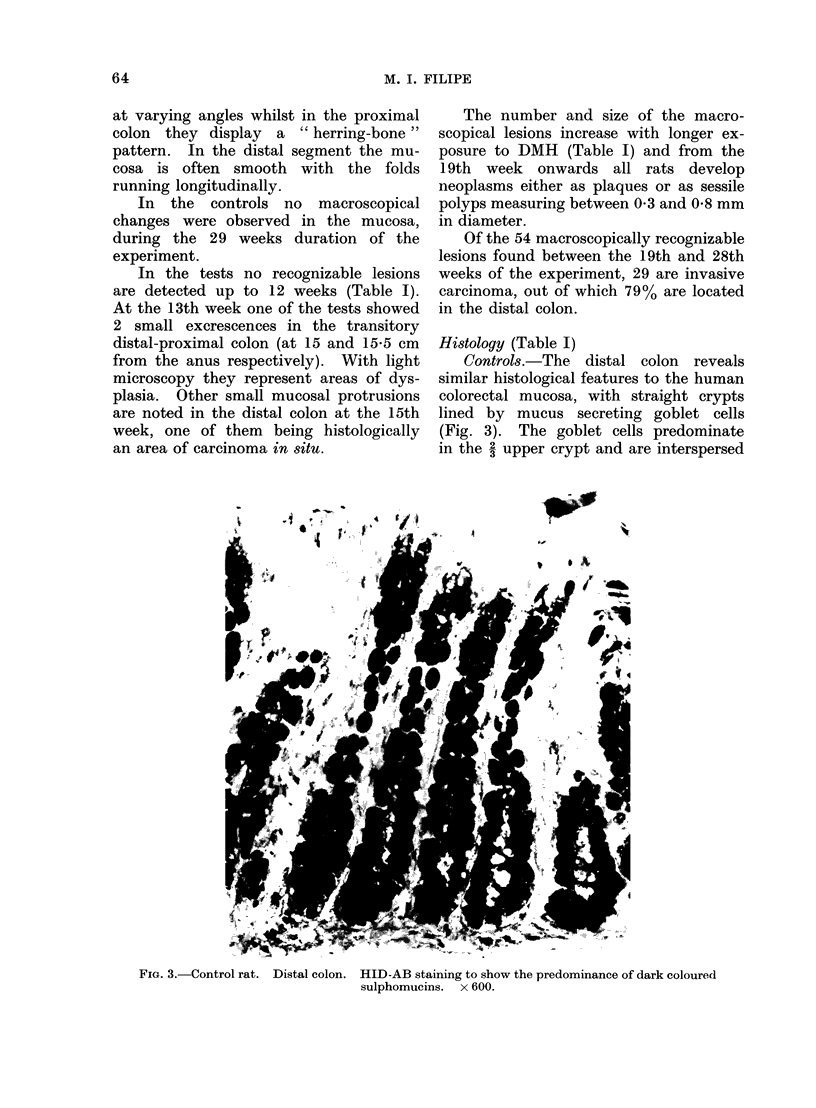

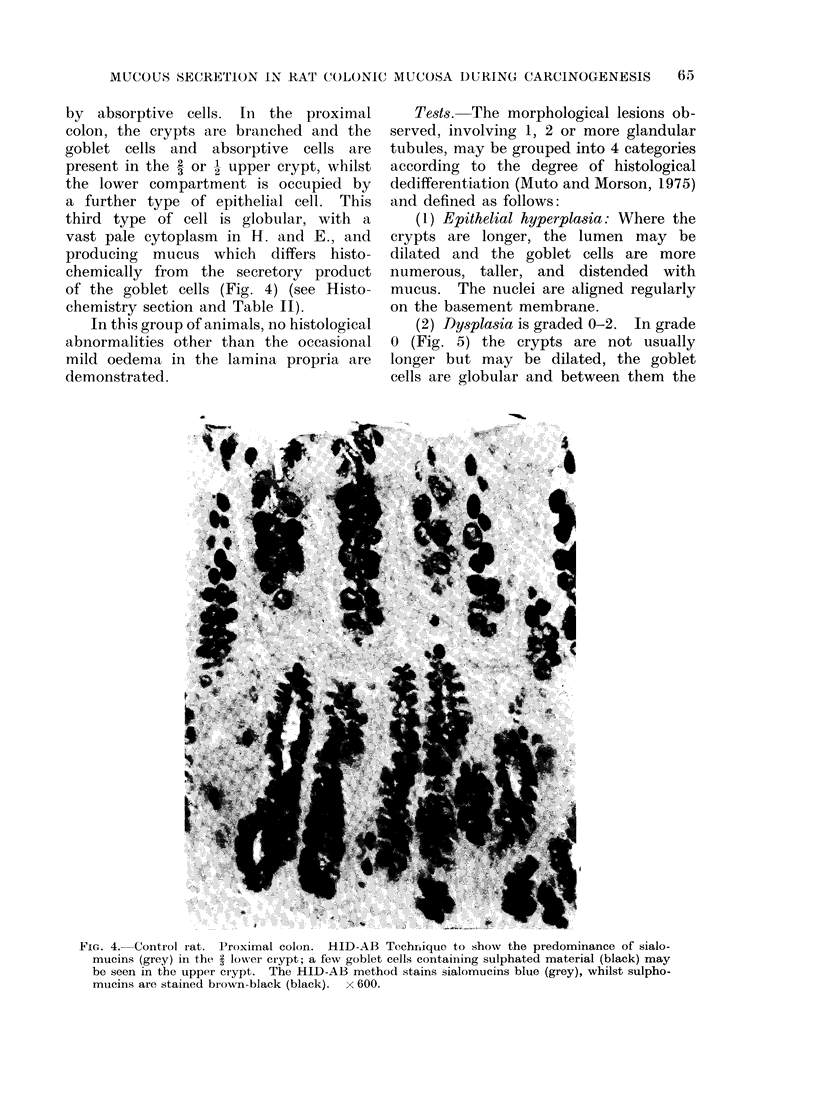

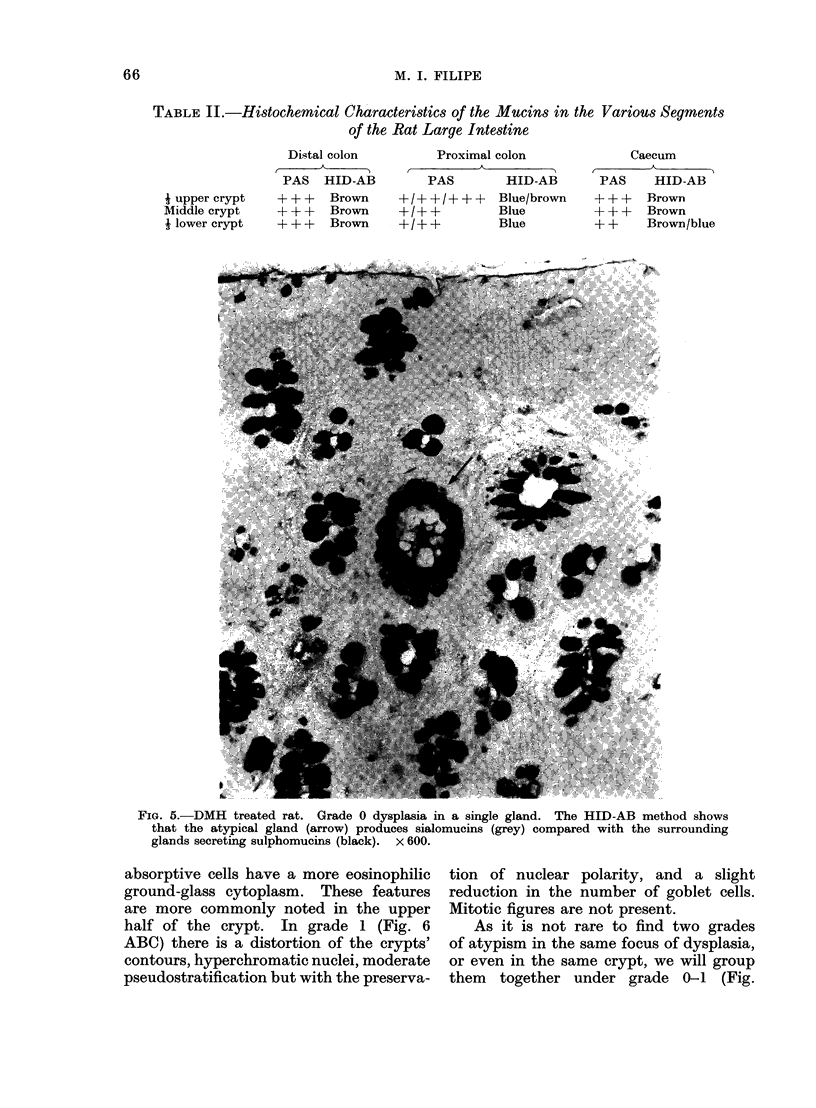

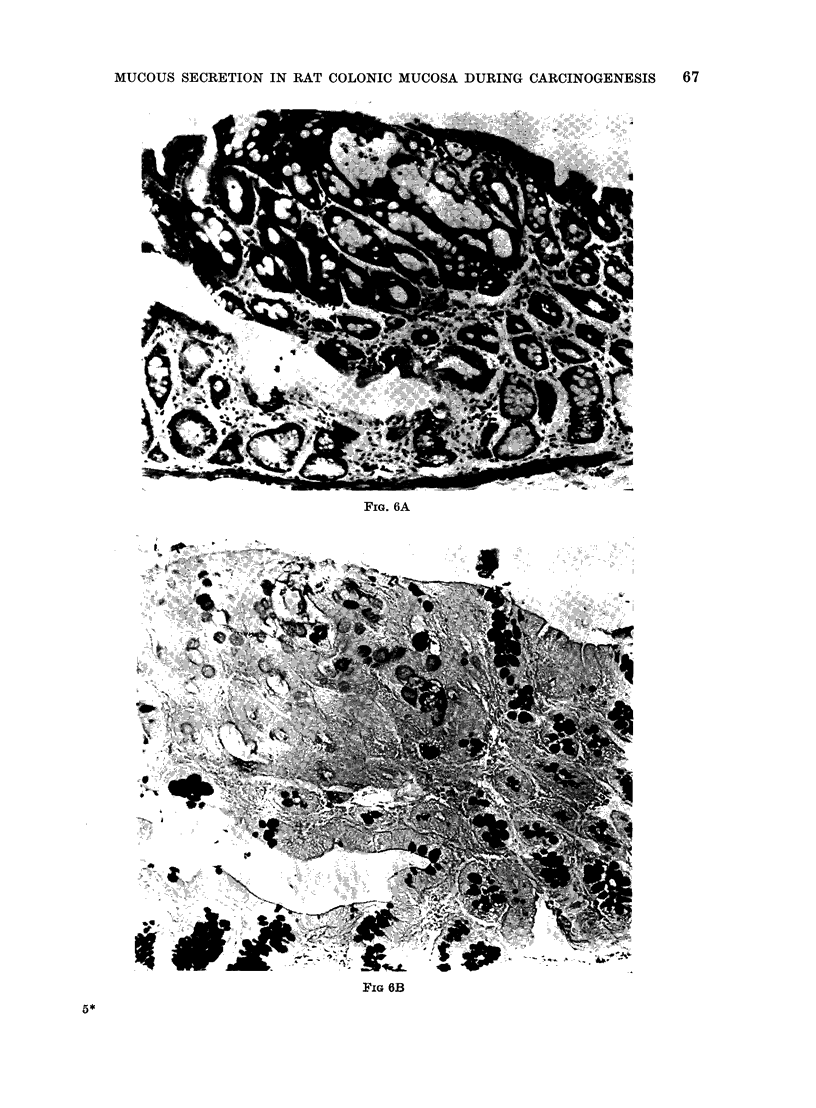

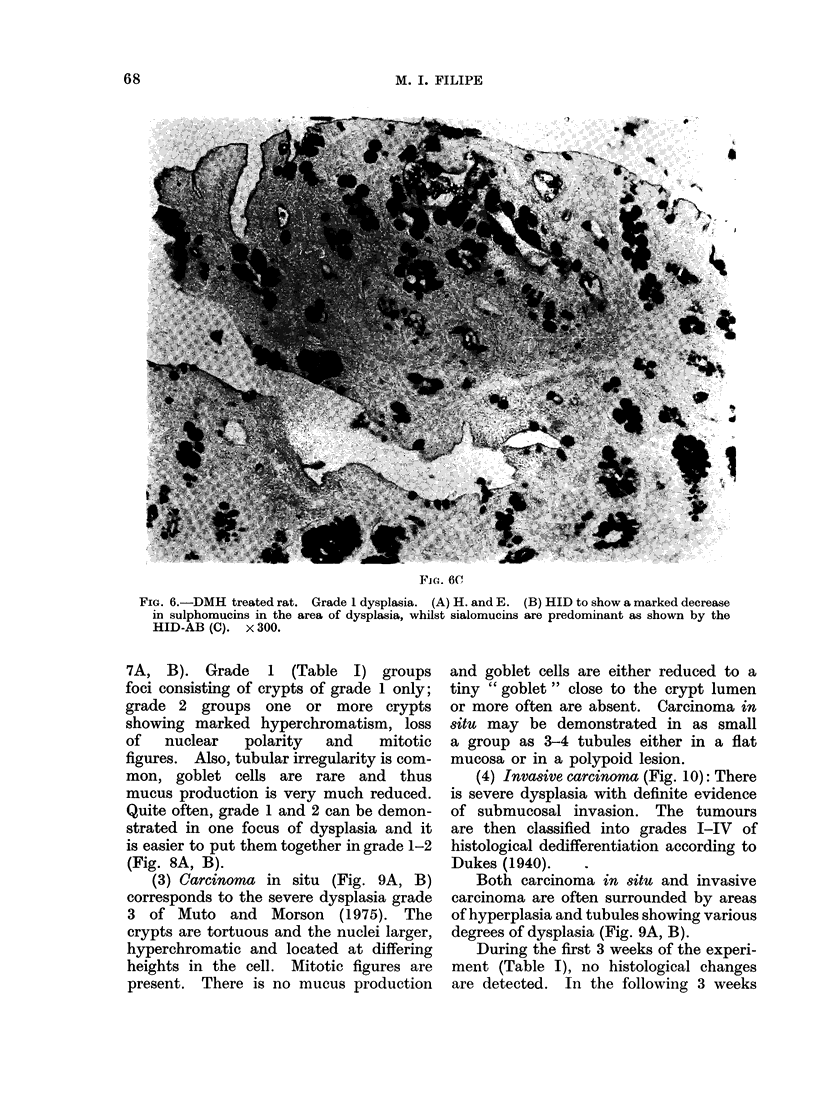

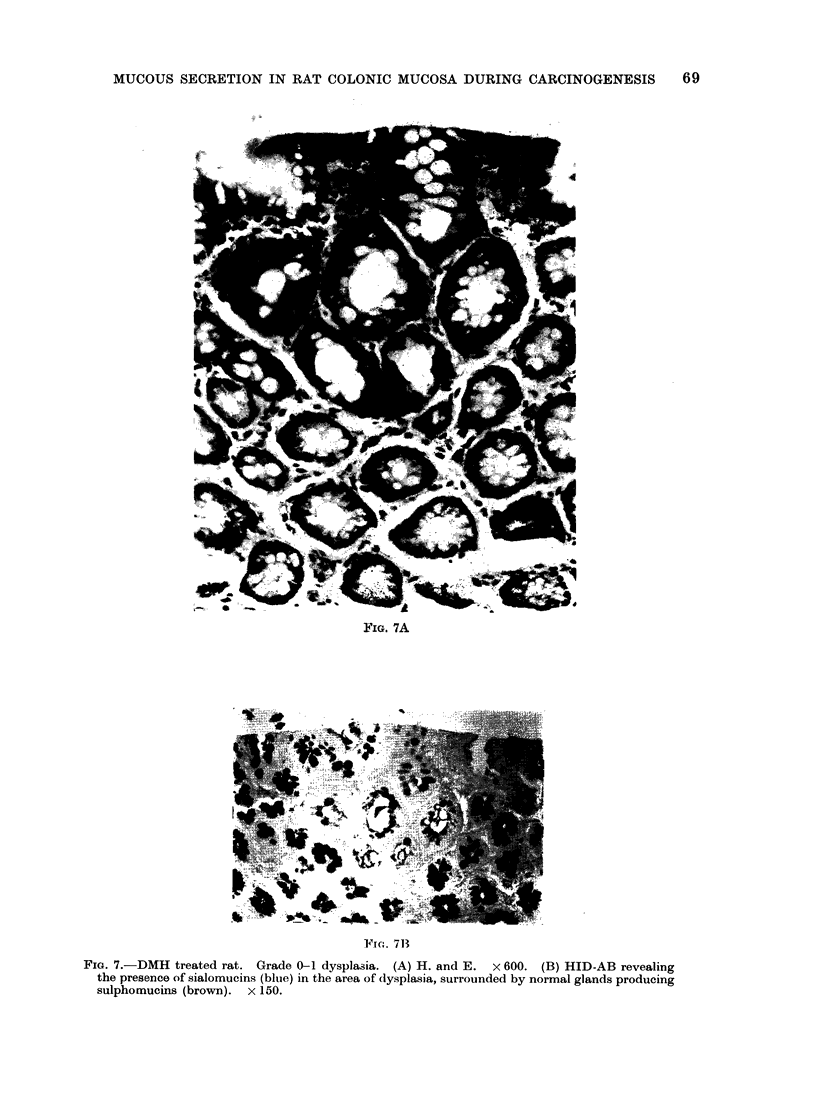

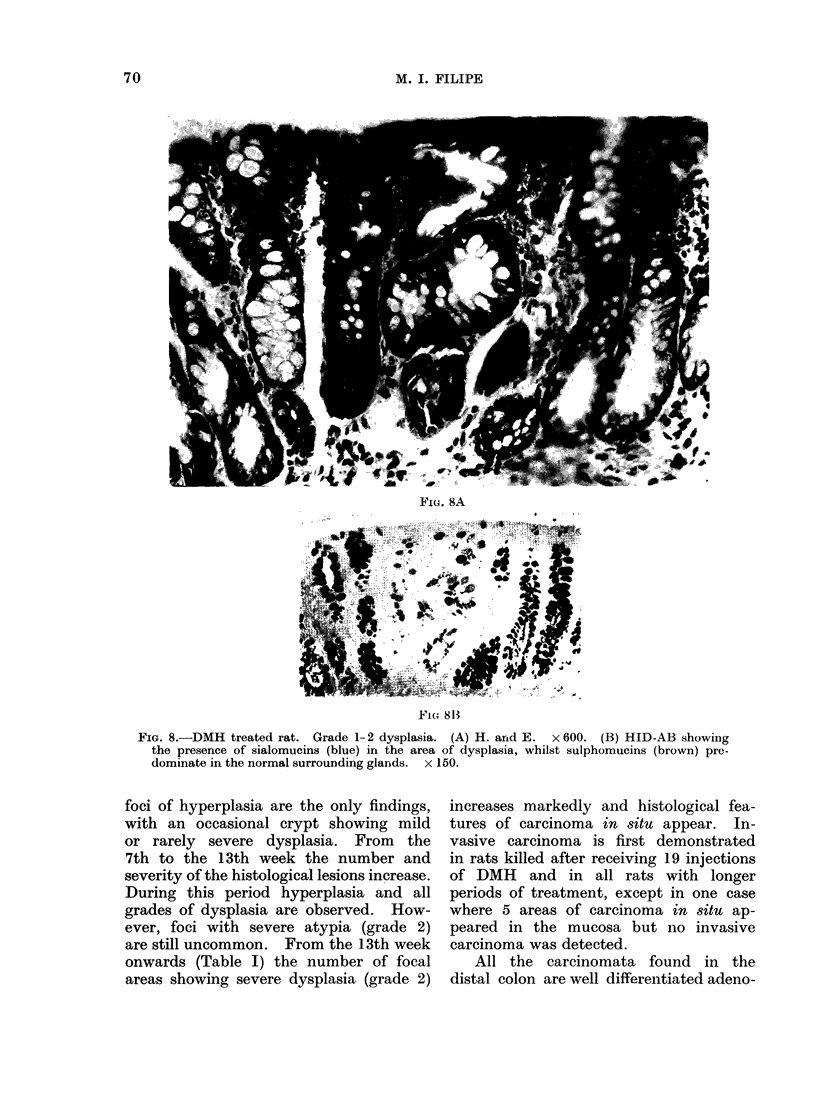

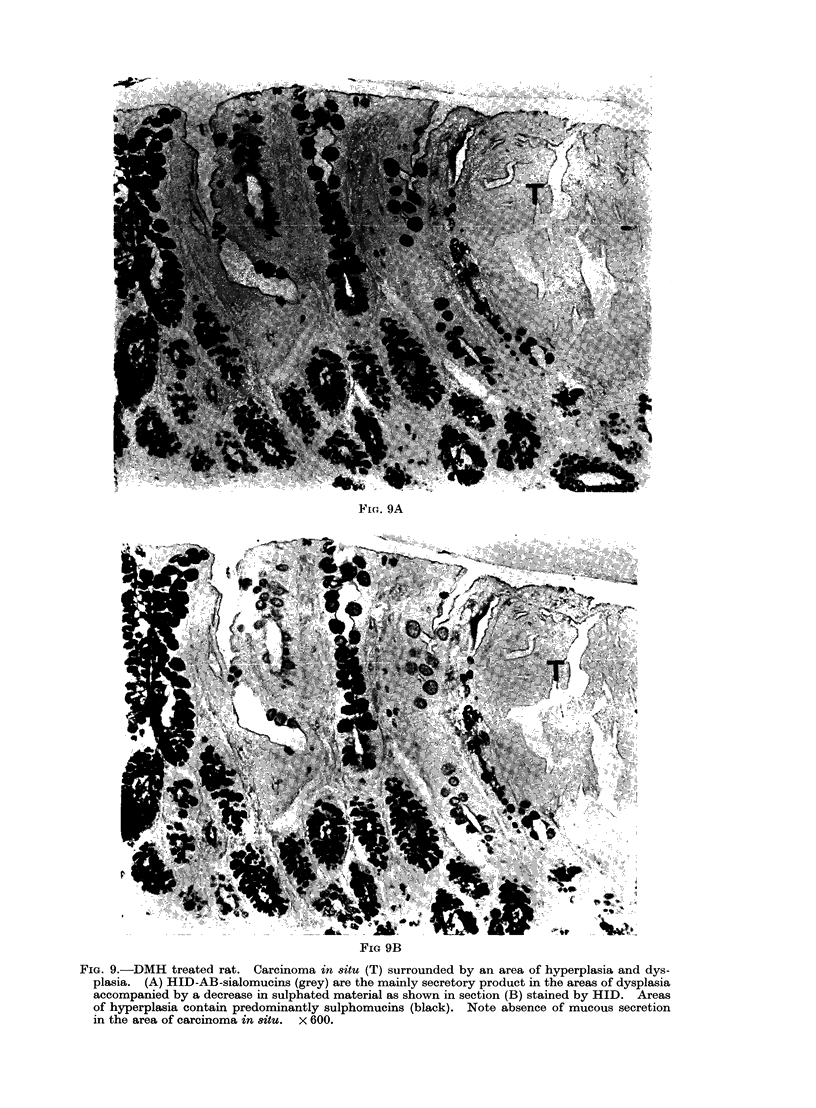

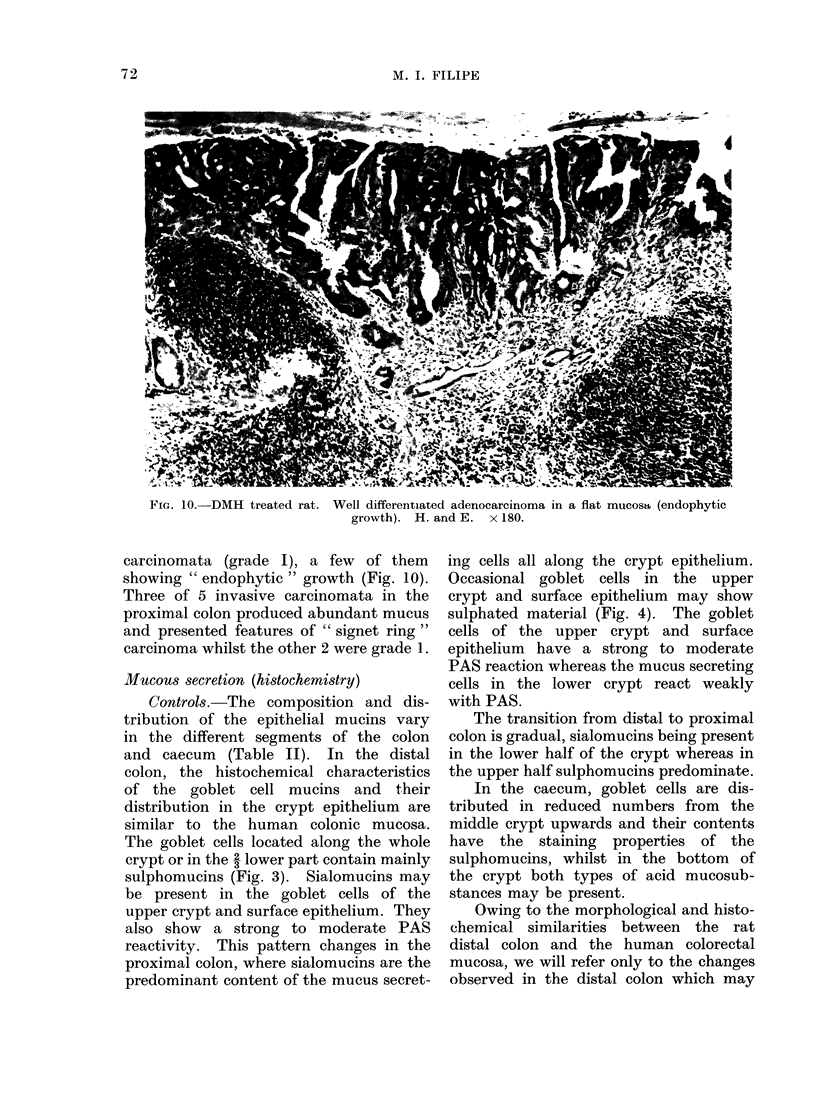

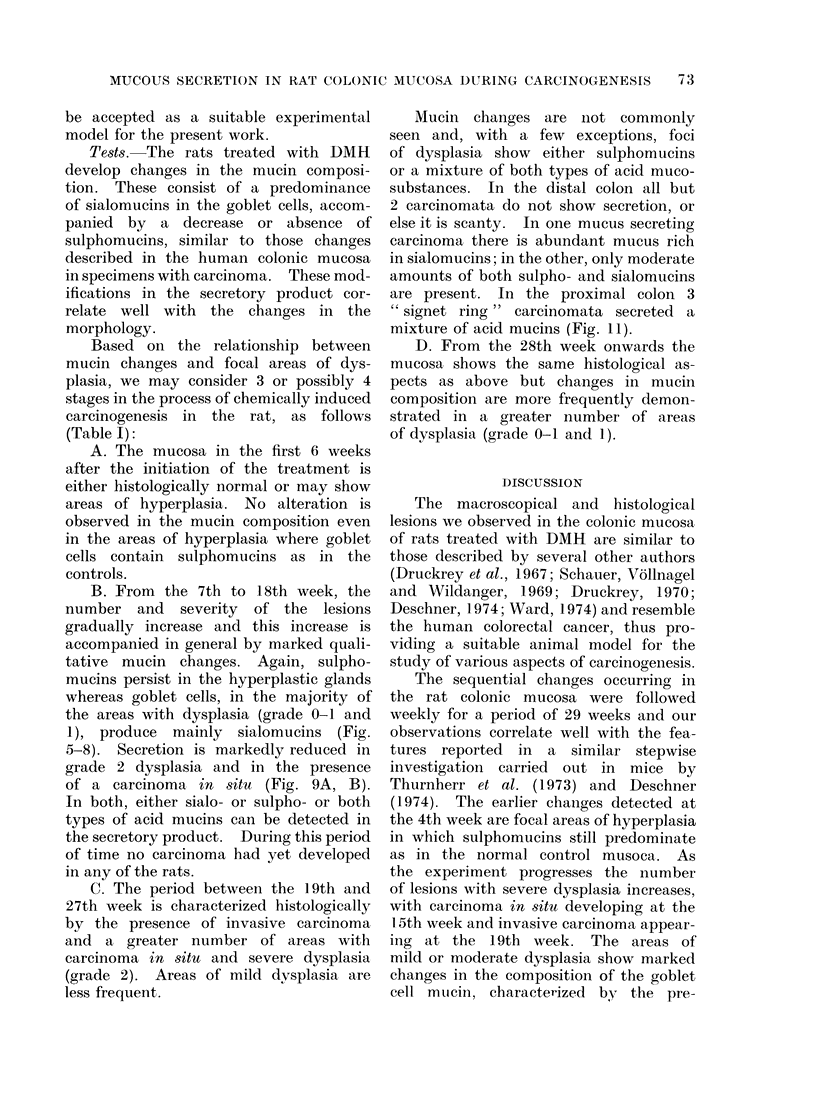

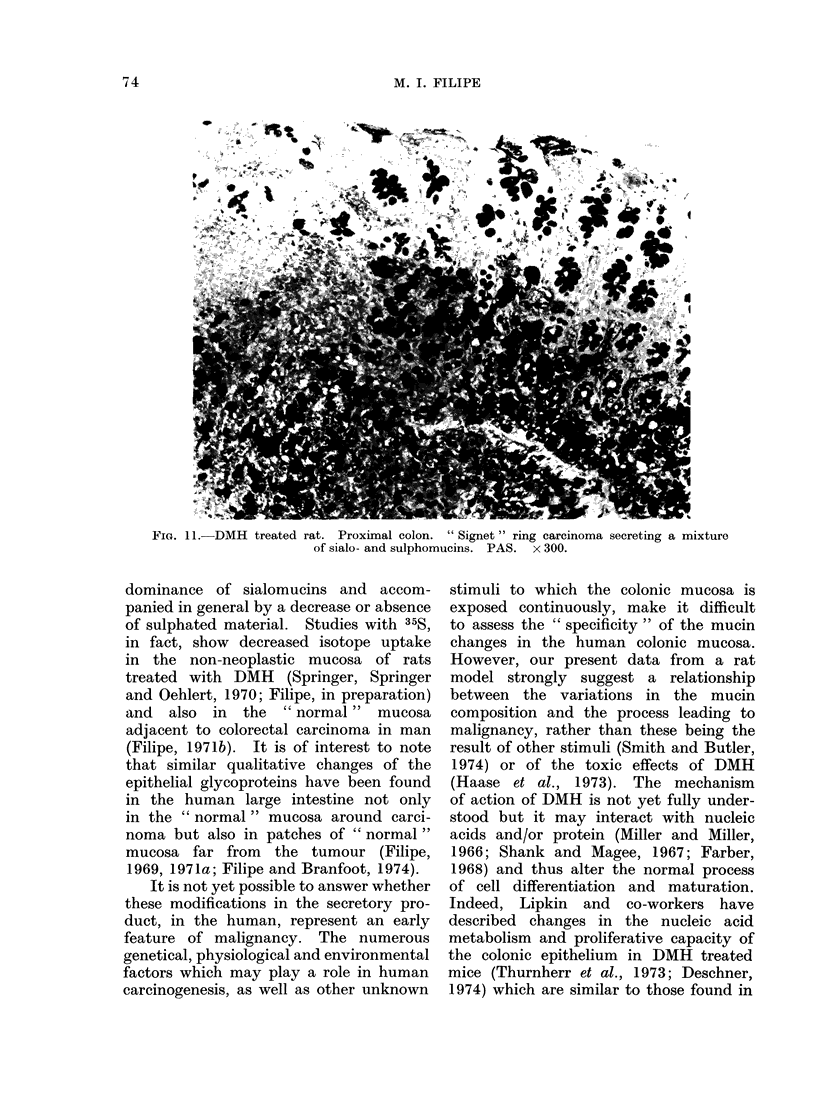

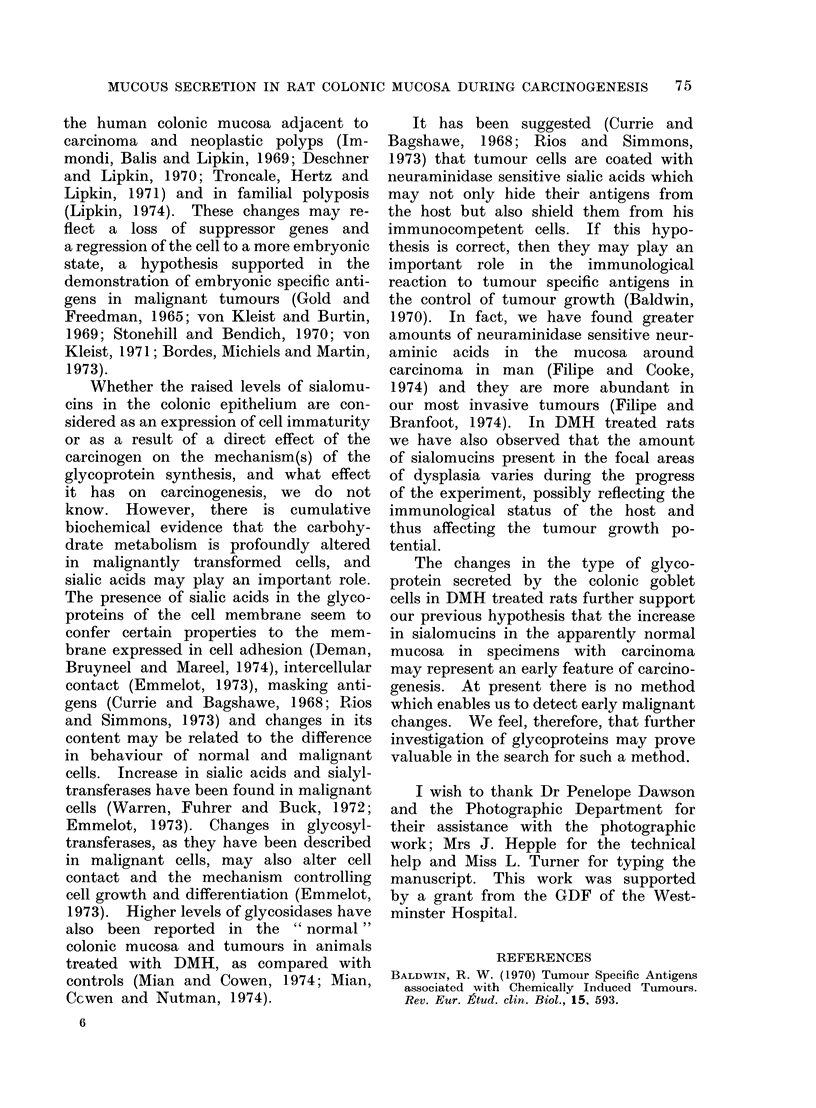

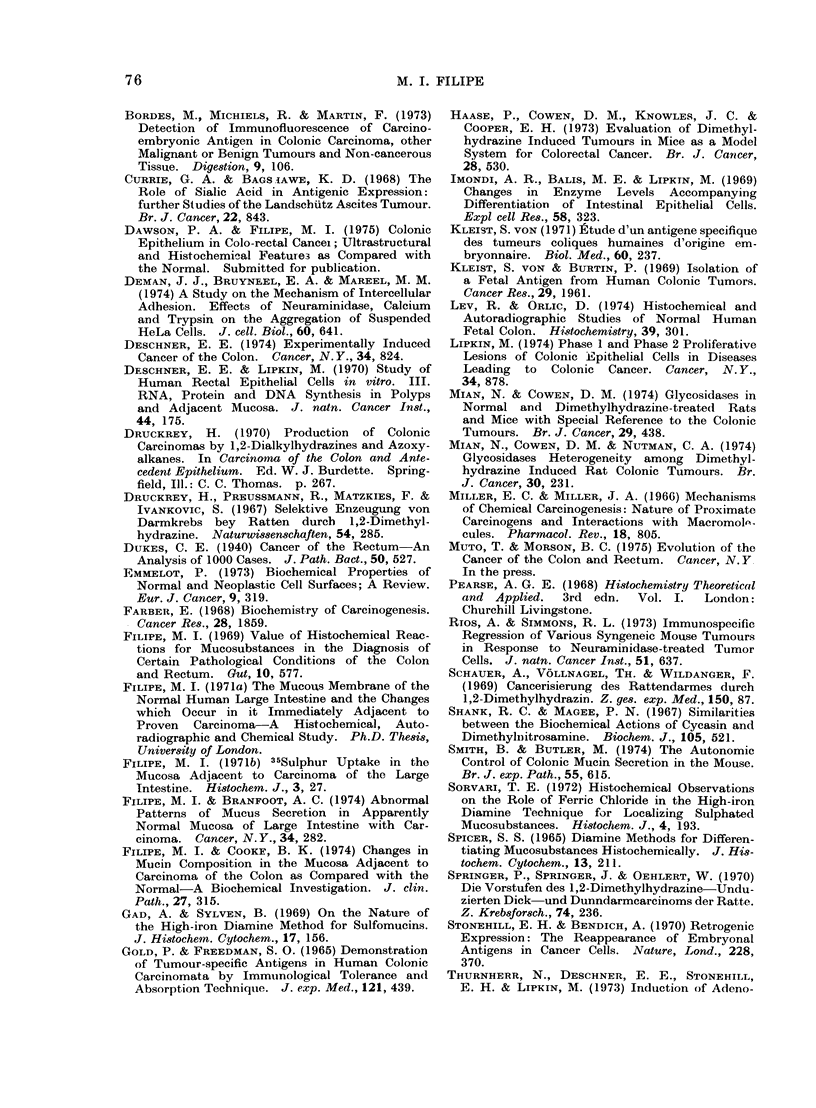

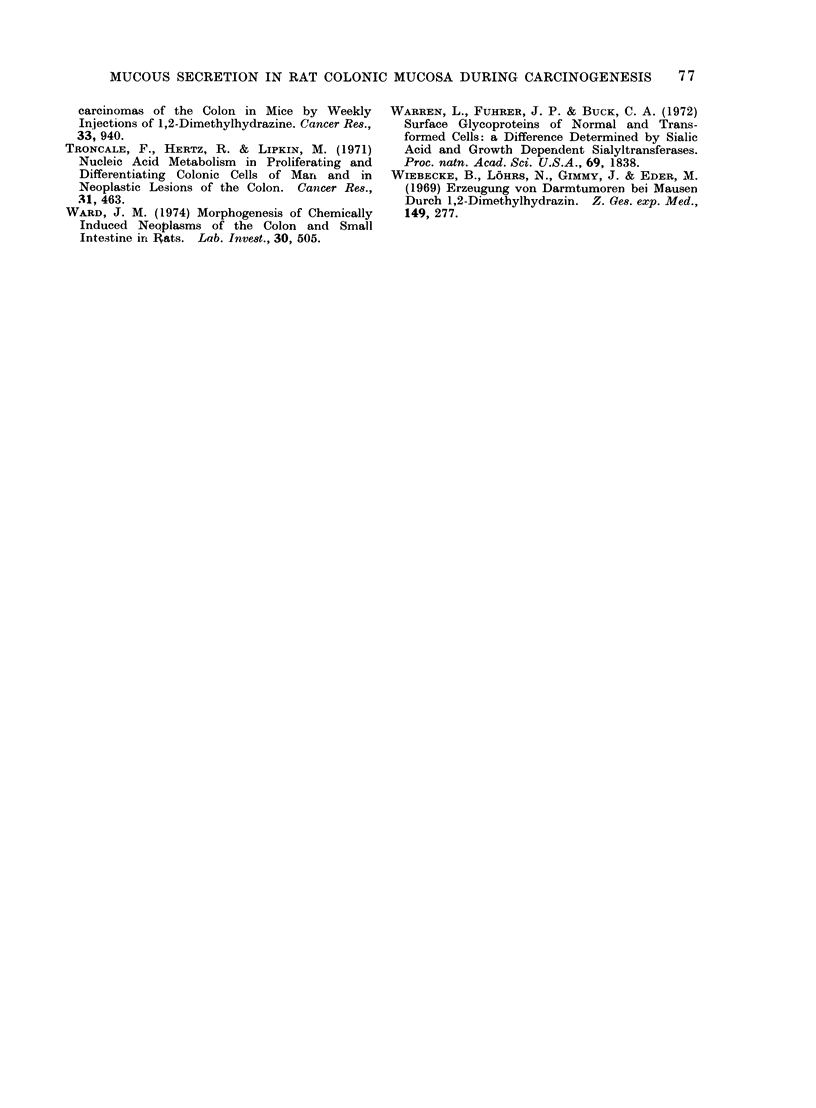


## References

[OCR_01033] Baldwin R. W. (1970). Tumour specific antigens associated with chemically induced tumours.. Rev Eur Etud Clin Biol.

[OCR_01042] Bordes M., Michiels R., Martin F. (1973). Detection by immunofluorescence of carcinoembryonic antigen in colonic carcinoma, other malignant or benign tumours, and non-cancerous tissues.. Digestion.

[OCR_01049] Currie G. A., Bagshawe K. D. (1968). The role of sialic acid in antigenic expression: further studies of the Landschütz ascites tumour.. Br J Cancer.

[OCR_01061] Deman J. J., Bruyneel E. A., Mareel M. M. (1974). A study on the mechanism of intercellular adhesion. Effects of neuraminidase, calcium, and trypsin on the aggregation of suspended HeLa cells.. J Cell Biol.

[OCR_01072] Deschner E. E., Lipkin M. (1970). Study of human rectal epithelial cells in vitro. III. RNA, protein, and DNA synthesis in polyps and adjacent mucosa.. J Natl Cancer Inst.

[OCR_01096] Emmelot P. (1973). Biochemical properties of normal and neoplastic cell surfaces; a review.. Eur J Cancer.

[OCR_01101] Farber E. (1968). Biochemistry of carcinogenesis.. Cancer Res.

[OCR_01119] Filipe M. I. (1971). 35 sulphur uptake in the mucosa adjacent to carcinoma of the large intestine.. Histochem J.

[OCR_01124] Filipe M. I., Branfoot A. C. (1974). Abnormal patterns of mucus secretion in apparently normal mucosa of large intestine with carcinoma.. Cancer.

[OCR_01130] Filipe M. I., Cooke K. B. (1974). Changes in composition of mucin in the mucosa adjacent to carcinoma of the colon as compared with the normal: a biochemical investigation.. J Clin Pathol.

[OCR_01105] Filipe M. I. (1969). Value of histochemical reactions for mucosubstances in the diagnosis of certain pathological conditions of the colon and rectum.. Gut.

[OCR_01142] GOLD P., FREEDMAN S. O. (1965). DEMONSTRATION OF TUMOR-SPECIFIC ANTIGENS IN HUMAN COLONIC CARCINOMATA BY IMMUNOLOGICAL TOLERANCE AND ABSORPTION TECHNIQUES.. J Exp Med.

[OCR_01137] Gad A., Sylvén B. (1969). On the nature of the high iron diamine method for sulfomucins.. J Histochem Cytochem.

[OCR_01148] Haase P., Cowen D. M., Knowles J. C., Cooper E. H. (1973). Evaluation of dimethylhydrazine induced tumours in mice as a model system for colorectal cancer.. Br J Cancer.

[OCR_01155] Imondi A. R., Balis M. E., Lipkin M. (1969). Changes in enzyme levels accompanying differentiation of intestinal epithelial cells.. Exp Cell Res.

[OCR_01171] Lev R., Orlic D. (1974). Histochemical and radioautographic studies of normal human fetal colon.. Histochemistry.

[OCR_01182] Mian N., Cowen D. M. (1974). Glycosidases in normal and dimethylhydrazine-treated rats and mice with special reference to the colonic tumours.. Br J Cancer.

[OCR_01188] Mian N., Cowen D. M., Nutman C. A. (1974). Glycosidases heterogeneity among dimethylhydrazine induced rat colonic tumours.. Br J Cancer.

[OCR_01194] Miller E. C., Miller J. A. (1966). Mechanisms of chemical carcinogenesis: nature of proximate carcinogens and interactions with macromolecules.. Pharmacol Rev.

[OCR_01210] Rios A., Simmons R. L. (1973). Immunospecific regression of various syngeneic mouse tumors in response to neuraminidase-treated tumor cells.. J Natl Cancer Inst.

[OCR_01236] SPICER S. S. (1965). DIAMINE METHODS FOR DIFFERENTIALING MUCOSUBSTANCES HISTOCHEMICALLY.. J Histochem Cytochem.

[OCR_01220] Shank R. C., Magee P. N. (1967). Similarities between the biochemical actions of cycasin and dimethylnitrosamine.. Biochem J.

[OCR_01225] Smith B., Butler M. (1974). The autonomic control of colonic mucin secretion in the mouse.. Br J Exp Pathol.

[OCR_01230] Sorvari T. E. (1972). Histochemical observations on the role of ferric chloride in the high-iron diamine technique for localizing sulphated mucosubstances.. Histochem J.

[OCR_01241] Springer P., Springer J., Oehlert W. (1970). Die Vorstufen des 1,2-Dimethylhydrazin-induzierten Dick- und Dünndarmcarcinoms der Ratte.. Z Krebsforsch.

[OCR_01247] Stonehill E. H., Bendich A. (1970). Retrogenetic expression: the reappearance of embryonal antigens in cancer cells.. Nature.

[OCR_01253] Thurnherr N., Deschner E. E., Stonehill E. H., Lipkin M. (1973). Induction of adenocarcinomas of the colon in mice by weekly injections of 1,2-dimethylhydrazine.. Cancer Res.

[OCR_01263] Troncale F., Hertz R., Lipkin M. (1971). Nucleic acid metabolism in proliferating and differentiating colonic cells of man and in neoplastic lesions of the colon.. Cancer Res.

[OCR_01270] Ward J. M. (1974). Morphogenesis of chemically induced neoplasms of the colon and small intestine in rats.. Lab Invest.

[OCR_01275] Warren L., Fuhrer J. P., Buck C. A. (1972). Surface glycoproteins of normal and transformed cells: a difference determined by sialic acid and a growth-dependent sialyl transferase.. Proc Natl Acad Sci U S A.

[OCR_01282] Wiebecke B., Löhrs U., Gimmy J., Eder M. (1969). Erzeugung von Darmtumoren bei Mäusen durch 1,2-Dimethylhydrazin.. Z Gesamte Exp Med.

[OCR_01166] von Kleist S., Burtin P. (1969). Isolation of a fetal antigen from human colonic tumors.. Cancer Res.

[OCR_01161] von Kleist S. (1971). Etude d'un antigène spécifique de tumeurs coliques humaines d'origine embryonnaire.. Biol Med (Paris).

